# Recent Updates in the Treatment of Neurodegenerative Disorders Using Natural Compounds

**DOI:** 10.1155/2014/979730

**Published:** 2014-04-23

**Authors:** Mahmood Rasool, Arif Malik, Muhammad Saeed Qureshi, Abdul Manan, Peter Natesan Pushparaj, Muhammad Asif, Mahmood Husain Qazi, Aamer Mahmood Qazi, Mohammad Amjad Kamal, Siew Hua Gan, Ishfaq Ahmed Sheikh

**Affiliations:** ^1^Center of Excellence in Genomic Medicine Research (CEGMR), King Abdulaziz University, Jeddah, Saudi Arabia; ^2^Institute of Molecular Biology and Biotechnology, (IMBB), The University of Lahore, Lahore 54000, Pakistan; ^3^Department of Biochemistry, Allama Iqbal Medical College, Lahore 54000, Pakistan; ^4^Department of Biotechnology and Informatics, BUITEMS, Quetta, Pakistan; ^5^Center for Research in Molecular Medicine (CRiMM), The University of Lahore, Lahore 54000, Pakistan; ^6^Ontario Institute for Cancer Research, MaRS Centre, Toronto, Canada; ^7^King Fahd Medical Research Center (KFMRC), King Abdulaziz University, Jeddah, P.O. Box 80216, Jeddah 21589, Saudi Arabia; ^8^Human Genome Centre, School of Medical Sciences, Universiti Sains Malaysia, Kubang Kerian, Kelantan, Malaysia

## Abstract

Neurodegenerative diseases are characterized by protein aggregates and inflammation as well as oxidative stress in the central nervous system (CNS). Multiple biological processes are linked to neurodegenerative diseases such as depletion or insufficient synthesis of neurotransmitters, oxidative stress, abnormal ubiquitination. Furthermore, damaging of blood brain barrier (BBB) in the CNS also leads to various CNS-related diseases. Even though synthetic drugs are used for the management of Alzheimer's disease, Parkinson's disease, autism, and many other chronic illnesses, they are not without side effects. The attentions of researchers have been inclined towards the phytochemicals, many of which have minimal side effects. Phytochemicals are promising therapeutic agents because many phytochemicals have anti-inflammatory, antioxidative as well as anticholinesterase activities. Various drugs of either synthetic or natural origin applied in the treatment of brain disorders need to cross the BBB before they can be used. This paper covers various researches related to phytochemicals used in the management of neurodegenerative disorders.

## 1. Introduction

Various neurodegenerative (progressive loss of structure and/or function of neurons) disorders share many common features at both cellular and subcellular levels. Intracellular and extracellular changes could be observed in Alzheimer's, Parkinson's, Huntington's, and other neurodegenerative diseases. As far as cellular and subcellular biological events are concerned, the cytosol and endoplasmic reticulum are responsible for the synthesis of new structural and functional protein molecules. Mechanisms of translational as well as posttranslational modifications are highly complex and sophisticated in nature. Any polypeptide that fails to fold properly is directed to its degradation processes or known as autophagy and ubiquitin proteasome system [1, 2].

**Figure 1:**
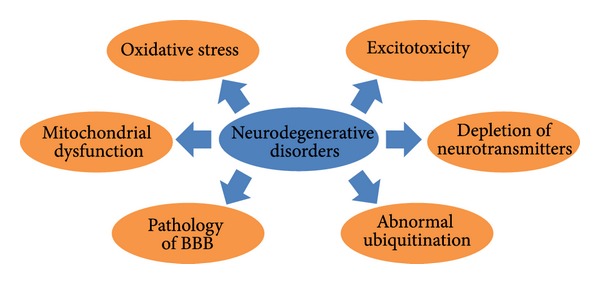
Various biological mechanisms contributing to neurodegenerative disorders.

Neurodegenerative disorders are usually characterized by accumulation of abnormal protein aggregation that leads to inflammation as well as oxidative stress in the central nervous system (CNS). Parkinson's disease (PD) and Alzheimer's disease (AD) are the most common disorders of nervous system caused by environmental and genetic influences [3–5]. It has been observed that various types of biological mechanisms are associated with neurodegenerative disorders such as oxidative stress, aggregates of proteins in neurons, depletion or in sufficient synthesis of neurotransmitters, degradation of neurotransmitters in the synaptic cleft due to the higher activity of enzymes, abnormal ubiquitination, mitochondrial dysfunction, and excitotoxicity of neurons as well as disarrangement or damage of the blood brain barrier (BBB) (Figure 1).

AD is characterized by cognitive decline, neuronal loss, neuronal inflammation, and neuronal death, which is also known as apoptosis and/or necroptosis. Moreover, aggregation of *β*-amyloid (A*β*) is one of the main features of AD. The formation of hyperphosphorylated Tau (microtubule-associated protein) in the neurons is also linked with AD. PD is a movement disorder which is characterized by abnormal aggregation of *α*-synuclein protein in the neurons [33]. Similarly, abnormal long polyglutamine (PolyQ) may lead to Huntington's disease [34].

Another important brain disorder related to CNS inflammation and characterized by learning and social disabilities with no definite pathogenesis is known as autism spectrum disorder (ASD). Multiple biochemical and molecular features could be observed for the neurodegeneration in the brain of ASD [35, 36] including oxidative stress [37, 38], activated astrocytes and microglia [39, 40], neuronal loss [35, 40], elevated levels of 8-oxo-guanosine [41], and development of proinflammatory cytokines [40, 42].

**Figure 2:**
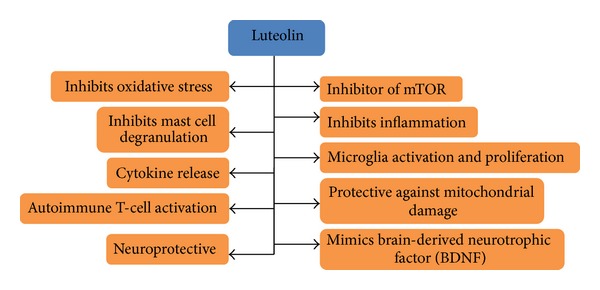
Luteolin (flavonoid) responsible for multiple biological functions.

Children with ASD tend to behave differently under stress or when exposed to certain foods, showing skin allergies [43]. Neurotensin with release of corticotrophin-releasing hormone under stressful conditions stimulates the microglia and mast cells leading to neurotoxicity and focal brain inflammation. In case of ASD, various pathological states could be observed but not in all ASD children including allergic symptoms, increased anti-brain protein autoantibodies, high anxiety, increased oxidative stress, and increased food intolerance while decreasing the levels of reduced glutathione, sulfation, and methylation [43]. Luteolin (a flavonoid) showed inhibitory effects on human mast cells that release tumor necrosis factor (TNF) [44]. Luteolin such as epigallocatechin gallate inhibits [45] mammalian target of rapamycin (mTOR) which stimulates the mast cells and microglia proliferation [46, 47] leading to the retardation of the release of TNF which could initiate apoptosis, necroptosis, and/or inflammation in the biological system. Various important biological actions of luteolin are illustrated (Figure 2) which may be helpful in children of ASD.

## 2. Blood Brain Barrier (BBB)

The blood brain barrier (BBB) is responsible for the regulation of small molecules (solutes) between the CNS and the blood circulation. Three different kinds of barriers could be observed where the central nervous system and blood interact; arachnoid barrier, blood-cerebrospinal fluid (CSF) barrier and the BBB. The neurons in the CNS signal by sending action potentials through which neurons interact in the biological system. The BBB had tight junctions between cells responsible for the reduction of flux mechanism through the paracellular pathway and intercellular cleft (physical barrier) and mediation of solute flux mechanisms (transport barrier) as well as enzyme metabolizing molecules (metabolic barrier). Moreover, the functions of barriers are equally operated in physiological and pathological states of BBB [48]. The tight junctions present between the astrocytes (part of the BBB) are composed of claudin and occludin proteins. Damage in these proteins or tight junctions can lead to the loss of BBB integrity with functional barrier loss [49, 50]. Various drugs, either synthetic or natural, may have their own mode of action but drugs used in the treatment of brain disorders have to cross the BBB to gain entry into the CNS, since structural and/or functional dysfunction in the BBB leads to inflammatory changes in the tissue such as movement of immune mediators in the brain, further contributing to the neurodegenerative process [51].

## 3. Inhibition of Cholinesterase Activity

Stimulating acetylcholine release in the brain region is one of the ways used in the treatment of neurodegenerative disorder such as AD that can further contribute to dementia and decline in higher cognitive function [52]. The pathological state of CNS particularly related to AD is characterized by neurofibrillary tangles, derangement of neurotransmitters in the neurons and synaptic cleft, and *β*-amyloids plaques all of which are related inflammatory mechanisms [53–55]. Both acetylcholinesterase (AChE) and butyrylcholinesterase (BChE) are responsible for the breakdown of acetylcholine in the synaptic region and low levels of acetylcholine has been found to be related to age-related disorders that leads to loss of cognitive ability [18, 56].

Reactive oxygen species (ROS) developed as a result of oxidative stress in the biological system can contribute to damage of biological macromolecules and as a result pathological state at cellular level can become more evident. Such pathogenic state plays a crucial role in the aging process [57]. Cholinesterase inhibitors are not commonly used in allopathic and current treatments do not lead to sufficient production of acetylcholine to help in the management of AD [18]. The research in the field of phytochemicals has developed into investigation of natural compounds responsible for antioxidative (Table 2) and antiaging properties that can also be useful for neurodegenerative disorders [18, 58]. It is important to stimulate the cholinergic receptors in the CNS or enhance the prolonged production of acetylcholine in the synaptic cleft with the help of such active constituents that could retard the activities of AChE and BChE in the neuronal system. When the inhibition of enzyme activities is 60% or more by the plants extracts, compounds are generally considered as strong inhibitors (Table 3) [59].

## 4. Anti-Inflammatory and Antioxidative Activites

Various medicinal plants have anti-inflammatory activities by inhibiting cyclooxygenase-1 (COX-1) that surrounds amyloids plaque in microglia. The accumulation of COX-1 enzyme in microglia in AD patients may be responsible for the local increase in oxidative stress and prostaglandin synthesis [10].* Ferula assafoetida*,* Syzygium aromaticum,* and* Zingiber officinalis* have previously been reported to have activity against COX-1 enzyme [10].* F. assafoetida* has previously been used as memory enhancer, antibacterial, antispasmodic, and antihelminthic in traditional medicines.* Z. officinalis* showed not only anti-COX-1 activity but also free radical scavenging activity that may be contributed to the presence of important phytochemicals such as gingerols and shogaols [10].

Sinapic acid (Brassicaceae) shows anti-inflammatory activity and can act as a neuroprotective agent by decreasing the levels of A*β* and by protecting neuronal cell death [15]. On the other hand,* Emblica officinalis* may be used in the treatment of mental disorders and as anti-inflammatory agent [60]. Several natural polyphenols such as vitamins, flavonoids, phenolic acids, and other polyphenols including thymol, ellagic acid, and eugenol have antioxidant properties and may be used for neurodegenerative diseases as promising therapeutic agents (Tables 1 and 3).

## 5. Computational Approaches towards Neurodegenerative Disorders

With the advancements in computational fields, particularly in the field of bioinformatics, the understanding of biological system at molecular level has improved drastically. The action of enzymes with their substrates, the synthesis of proteins, degradation of various biological macromolecules, ubiquitination, and many other processes could be observed with various computational programs including* in silico* molecular docking strategies. The normal homeostasis including metabolic equilibrium associated with many complex biological mechanisms under the supervision of autonomic nervous system as well as prediction for pathological state and possible therapeutic suggestions.

Jeyam et al. [7] used the* in silico* techniques for the understanding of molecular behavior of some traditional medicines for the management of PD. The loss of dopamine is considered as prominent feature of PD. Currently, levodopa (L-Dopa) is given in the form of supplementation for the management of PD. Catecholamine-O-methyltransferase (COMT), an enzyme, is responsible for the metabolism and conversion of L-Dopa into 3-O-methyl dopa. Hence, the inhibition of COMT may be one of the important ways to treat the disorder. Considering this way of treatment, the neuroprotective phytocompounds were evaluated using* in silico* studies [7]. Phytochemicals such as baicalin, stigmasterol, emodin, curcumin, wogonin, and eriodictyol were found to be having binding energies of approximately −7 kcal/mol which was similar to talcapone (synthetic drug to enhance the levodopa treatment) indicating that amentoflavone from* Ginkgo biloba* and ginsenoside from* Panax ginseng* are perceived as very good inhibitors for COMT as well as good adjuvants for L-dopa management. Kuhn and Kollman [61] studied and calculated the free energy activation of COMT considering the molecular dynamics of this enzyme. Moreover, Lee and Kim [62] investigated human COMT for designing anti-PD drug by using the ligand docking and comparative homology modeling.

Ayurveda medication has been evaluated for schizophrenia using* in silico* techniques [8]. Schizophrenia is associated with misbalancing of various chemicals of the brain involving the glutamate and dopamine. Studies on schizophrenia indicated that patients have abnormalities in brain structure such as decreased size of certain brain regions, enlargement of fluid-filled cavities, and less metabolic activities. Moreover, patients have delusions and hallucinations. From the Indian medication, three plants (*Rauvolfia serpentina*,* Withania somnifera,* and Mandukparni) were selected for the investigation of their role in the management of schizophrenia by using the tools of bioinformatics. The active molecules from these plants were docked with RGS-4 protein (regulator for G protein signaling-4) considered to be responsible for schizophrenia. The docking of RGS-4 protein with the combinations of reserpine, withanolide, and asiaticoside from* Rauvolfia serpentina*,* Withania somnifera,* and Mandukparni, respectively, showed that such combination therapy could be helpful in the management of schizophrenia [8].

## 6. Conclusion

In future, phytochemicals could be used as promising therapeutic agents for neurodegenerative disorders due to their anti-inflammatory and antioxidative as well as anticholinesterase activities. The neurodegenerative disorders such as AD, PD, Huntington's, and others share common features at cellular and subcellular levels as well as sharing mostly common molecular signaling pathways that may lead to apoptosis, necroptosis, and inflammation. Overall phytochemicals provide promising alternatives to current therapies for neurodegenerative disorders.

## Figures and Tables

**Table 1:** Role of various plants and their active constituents in brain disorders.

Plant	Active Compounds	Disorder	References
*Adhatoda vasica *	Vasicine, vasicol, vasicinol, arachidic, cerotic, linoleic and oleic acids	AD, PD	[6]
*Ginkgo biloba *	Amentoflavone	PD	[7]
*Mandukparni *	Asiaticoside	Schizophrenia	[8]
*Panax ginseng *	Ginsenoside	PD	[7]
*Rauvolfia serpentina *	Reserpine	Schizophrenia	[8]
*Withania somnifera *	Withaferin A, sitoindoside IX, physagulin D, withanoside IV, viscosalactone B	Schizophrenia	[8]

**Table 2:** Plants with antioxidant properties which could be applied in the therapy used in neurodegenerative diseases.

Plants	Active Compounds	References
*Abrus precatorius *	Glycyrrhizin, precol, abrol, gallic acid, abrine	[9]
*Acorus calamus *	*α*-asarone, *β*-asrone, eugenol	[6]
*Adhatoda vasica *	Vasicine, vasicol, vasicinol, arachidic, cerotic, linoleic, oleic acids	[6]
*Anogeissus leiocarpus *	Castalagin, flavogallonic acid	[9]
*Emblica officinalis *	Emblicanins A, B, punigluconin, pedunculagin, punicafolin, ellagic acid, gallic acid	[10]
*Entandrophragma angolense *	7*α*-obacunyl acetate, cycloartane	[9]
*Khaya senegalensis *	Khayseneganin, luteolin, catechin	[9]
*Medicago sativa *	Soysaponin I, azukisaponin V	[6]
*Mentha spicata *	Spearmint oil, *α*, *β*-pinene, carvone, linalool, limonene	[6]
*Myrtus communis *	*α*-pinene, 1, 8-cineole, limonene	[6]
*Pavetta crassipes *	Quercetin	[9]
*Piper nigrum *	Piperine	[11]
*Salvia triloba *	Rosmarinic acid, ferulic acid, luteolin, quercetin	[11]
*Sonchus eruca *	Alkaloids, flavonoids, tannins, saponins	[12]
*Terminalia arjuna *	Arjunic acid, arjunolic acid, gallic acid, ellagic acid, proanthocyanidins	[10]
*Terminalia chebula *	Arjungenin, chebulosides, gallic acid, ellagic acid, luteolin, tannic acid, luteic acid, chebulic acid	[10]
*Tribulus terrestris *	Neohecogenin, *β*-D-galactopyranside	[6]
*Withania coagulaus *	Coagulin, withanolide, withaferin A	[6]
*Withania somnifera *	Withaferin A, sitoindoside IX, physagulin D, withanoside IV, viscosalactone B	[6]

**Table 3:** List of plants having antioxidative and anticholinesterase activity.

Plant	Active compounds	Properties	References
*Acorus calamus *	*α*-asarone, *β*-asrone, eugenol	Antioxidative, anticholinesterase	[13]
*Adhatoda vasica *	Vasicine, vasicol, vasicinol, arachidic, cerotic, linoleic, oleic acids	Anticholinesterase	[10]
*Bacopa monnieri *	Bacoside, brahmin, herpestine, d-mannitol, luteolin, apigenin	Anticholinesterase	[14]
*Brassica species *	Brassicasterol, sinapic acid, sinapine	Anti-inflammatory, neuroprotective, anticholinesterase	[15–17]
*Buddleja salviifolia *	Phenols, flavonoids, proanthocyanidins	Antioxidative, anticholinesterase	[18]
*Chamaecrista mimosoides *	Phenols, flavonoids, proanthocyanidins	Antioxidative, anticholinesterase	[18]
*Corydalis species *		AChE inhibition	[19]
*Corydalis ternate *	Protopine	Anticholinesterase, antiamnesic	[20]
*Cymbopogon schoenanthus *	Piperitone, 2-carene	Antioxidative, anticholinesterase, antimicrobial	[21]
*Ferula assafoetida *	Cadinene, eremophilene	Anti-COX-1	[10]
*Ginkgo biloba *	Ginkgetin, ginkoglides-A, B	Anticholinesterase	[14]
*Myricaria elegans *	Crude extract	Anticholinesterase, antilipooxygenase	[22]
*Nardostachys jatamansi *	Angelicin, *β*-eudesmol, calarene, calarenol, elemol, nardol, oroselol	Antioxidative, anticholinesterase	[13]
*Origanum ehrenbergii *	Carvacrol, thymol	Antioxidative, anti-inflammatory	[23]
*Origanum syriacum *	Carvacrol, thymol	Antioxidative, anti-inflammatory, anticholinesterase	[23]
*Peganum harmala *	Norharmane, harmine, harmalol	Anticholinesterase	[10]
*Piper nigrum *	Piperine	Antioxidative, anticholinesterase	[11]
*Ptychopetalum olacoides *	Lupeol, *α*, *β*-pinene	Anticholinesterase	[24]
*Salvia lavandulaefolia *	Essential oil, terpenes	Anticholinesterase	[25]
*Salvia miltiorrhiza*	Diterpenoid	Anticholinesterase	[26]
*Salvia miltiorrhiza *	Terpenes, tanshinones	Anticholinesterase	[26, 27]
*Salvia officinalis *	Polyphenols	Antioxidative, anticholinesterase	[28, 29]
*Salvia plebeian *	Essential oil	Antioxidative	[30]
*Salvia tiliifolia *	Phenols, flavonoids, proanthocyanidins	Antioxidative, inhibition of cholinesterase	[18]
*Salvia triloba *	Rosmarinic acid, ferulic acid, luteolin, quercetin	Antioxidative, anticholinesterase	[11]
*Schotia brachypetala (root) *	Phenols, flavonoids, proanthocyanidins	Antioxidative, anticholinesterase	[18]
*Schotia brachypetala (bark) *	Phenols, flavonoids, proanthocyanidins	Antioxidative, anticholinesterase	[18]
*Syzygium aromaticum *	Eugenol, trans-*β*-caryophyllene, *α*-humulene	Anti-COX-1	[10]
*Tabernaemontana divaricata *	Voafinidine, lupeol, *α*-amyrin, *β*-sitosterol	Anticholinesterase	[31]
*Terminalia chebula *	Penta-O-galloyl-*β*-D-glucose	Anticholinesterase	[32]
*Zingiber officinale *	Gingerol, shogaol, zingerone	Anti-COX-1	[10]

## References

[1] Wong E, Cuervo AM (2010). Autophagy gone awry in neurodegenerative diseases. *Nature Neuroscience*.

[2] Ciechanover A (2006). The ubiquitin proteolytic system: from a vague idea, through basic mechanisms, and onto human diseases and drug targeting. *Neurology*.

[3] Aruoma OI, Bahorun T, Jen L-S (2003). Neuroprotection by bioactive components in medicinal and food plant extracts. *Mutation Research*.

[4] Jenner P, Olanow CW (1998). Understanding cell death in Parkinson’s disease. *Annals of Neurology*.

[5] Behl C (1999). Alzheimer’s disease and oxidative stress: implications for novel therapeutic approaches. *Progress in Neurobiology*.

[6] Hussain I, Khan N, Khan H (2010). Screening of anti-oxidant activities of selected medicinal plants. *World Applied Sciences Journal*.

[7] Jeyam M, Karthika GRR, Poornima V, Sharanya M (2012). Molecular understanding and in silico validation of traditional medicines for Parkinson's disease. *Asian Journal of Pharmaceutical and Clinical Research*.

[8] Bagchi P, Kar A, Vinobha CS (2009). Establishing an in-silico ayurvedic medication towards treatment of Schizophrenia. *International Journal of Systems Biology*.

[9] Olutayo O, Doyinsola I, Simon O, Abayomi O, Thomas S (2011). Phytochemical and antioxidant properties of some Nigerian medicinal plants. *American Journal of Scientific and Industrial Research*.

[10] Ali SK, Hamed AR, Soltan MM (2013). In-vitro evaluation of selected Egyptian traditional herbal medicines for treatment of Alzheimer disease. *BMC Complementary and Alternative Medicine*.

[11] Mahdy K, Shaker O, Wafay H, Nassar Y, Hassan H, Hussein A (2012). Effect of some medicinal plant extracts on the oxidative stress status in Alzheimer's disease induced in rats. *European Review for Medical and Pharmacological Sciences*.

[12] Ullah R, Khader JA, AbdEIslam NM (2013). Antioxidant activity of different crude fractions of *Sonchus eruca*. *Life Science Journal*.

[13] Ahmed F, Chandra NS, Urooj A, Rangappa KS (2009). *In vitro* antioxidant and anti-cholinesterase activity of Acorus calamus and Nardostachys jatamansi rhizomes. *Journal of Pharmacy Research*.

[14] Das A, Shanker G, Nath C, Pal R, Singh S, Singh HK (2002). A comparative study in rodents of standardized extracts of Bacopa monniera and Ginkgo biloba anti-cholinesterase and cognitive enhancing activities. *Pharmacology Biochemistry and Behavior*.

[15] Lee HE, Kim DH, Park SJ (2012). Neuroprotective effect of sinapic acid in a mouse model of amyloid beta(1-42) protein-induced Alzheimer's disease. *Pharmacology Biochemistry and Behavior*.

[16] Vanmierlo T, Popp J, Kölsch H (2011). The plant sterol brassicasterol as additional CSF biomarker in Alzheimer’s disease. *Acta Psychiatrica Scandinavica*.

[17] Dong HK, Byung HY, Kim Y-W (2007). The seed extract of Cassia obtusifolia ameliorates learning and memory impairments induced by scopolamine or transient cerebral hypoperfusion in mice. *Journal of Pharmacological Sciences*.

[18] Adewusi EA, Moodley N, Steenkamp V (2011). Antioxidant and acetylcholinesterase inhibitory activity of selected southern African medicinal plants. *South African Journal of Botany*.

[19] Adsersen A, Gauguin B, Gudiksen L, Jäger AK (2006). Screening of plants used in Danish folk medicine to treat memory dysfunction for acetylcholinesterase inhibitory activity. *Journal of Ethnopharmacology*.

[20] Kim SR, Hwang SY, Jang YP (1999). Protopine from Corydalis ternata has anticholinesterase and antiamnesic activities. *Planta Medica*.

[21] Khadri A, Neffati M, Smiti S (2010). Antioxidant, anti-acetylcholinesterase and antimicrobial activities of *Cymbopogon schoenanthus* L. Spreng, (lemon grass) from Tunisia. *LWT Food Science and Technology*.

[22] Ahmad W, Ahmad B, Ahmad M, Iqbal Z, Nisar M, Ahmad M (2003). *In vitro* inhibition of acetylcholinesterase, butyrylcholinesterase and lipoxygenase by crude extract of Myriacaria elegans Royle. *Journal of Biological Sciences*.

[23] Loizzo MR, Menichini F, Conforti F (2009). Chemical analysis, antioxidant, antiinflammatory and anticholinesterase activities of Origanum ehrenbergii Boiss and Origanum syriacum L. essential oils. *Food Chemistry*.

[24] Siqueira IR, Fochesatto C, da Silva AL (2003). *Ptychopetalum olacoides*, a traditional Amazonian “nerve tonic”, possesses anticholinesterase activity. *Pharmacology Biochemistry and Behavior*.

[25] Perry NSL, Houghton PJ, Theobald A, Jenner P, Perry EK (2000). *In vitro* inhibition of human erythrocyte acetylcholinesterase by *Salvia lavandulaefolia* essential oil and constituent terpenes. *Journal of Pharmacy and Pharmacology*.

[26] Ren Y, Houghton PJ, Hider RC, Howes M-JR (2004). Novel diterpenoid acetylcholinesterase inhibitors from *Salvia miltiorhiza*. *Planta Medica*.

[27] Orhan I, Aslan M (2009). Appraisal of scopolamine-induced antiamnesic effect in mice and *in vitro* antiacetylcholinesterase and antioxidant activities of some traditionally used Lamiaceae plants. *Journal of Ethnopharmacology*.

[28] Lu Y, Yeap Foo L (2001). Antioxidant activities of polyphenols from sage (Salvia officinalis). *Food Chemistry*.

[29] Orhan I, Kartal M, Naz Q (2007). Antioxidant and anticholinesterase evaluation of selected Turkish Salvia species. *Food Chemistry*.

[30] Weng XC, Wang W (2000). Antioxidant activity of compounds isolated from *Salvia plebeia*. *Food Chemistry*.

[31] Chattipakorn S, Pongpanparadorn A, Pratchayasakul W, Pongchaidacha A, Ingkaninan K, Chattipakorn N (2007). *Tabernaemontana divaricata* extract inhibits neuronal acetylcholinesterase activity in rats. *Journal of Ethnopharmacology*.

[32] Sancheti S, Sancheti S, Um B-H, Seo S-Y (2010). 1,2,3,4,6-penta-O-galloyl-*β*-d-glucose: a cholinesterase inhibitor from *Terminalia chebula*. *South African Journal of Botany*.

[33] Lee VM-Y, Trojanowski JQ (2006). Mechanisms of Parkinson’s disease linked to pathological alpha-synuclein: new targets for drug discovery. *Neuron*.

[34] Bates G (2003). Huntingtin aggregation and toxicity in Huntington’s disease. *The Lancet*.

[35] Courchesne E, Pierce K, Schumann CM (2007). Mapping early brain development in autism. *Neuron*.

[36] Kern JK, Geier DA, Sykes LK, Geier MR (2013). Evidence of neurodegeneration in autism spectrum disorder. *Translational Neurodegeneration*.

[37] Chauhan A, Gu F, Essa MM (2011). Brain region-specific deficit in mitochondrial electron transport chain complexes in children with autism. *Journal of Neurochemistry*.

[38] Chauhan A, Audhya T, Chauhan V (2012). Brain region-specific glutathione redox imbalance in autism. *Neurochemical Research*.

[39] Morgan JT, Chana G, Pardo CA (2010). Microglial activation and increased microglial density observed in the dorsolateral prefrontal cortex in autism. *Biological Psychiatry*.

[40] Vargas DL, Nascimbene C, Krishnan C, Zimmerman AW, Pardo CA (2005). Neuroglial activation and neuroinflammation in the brain of patients with autism. *Annals of Neurology*.

[41] Sajdel-Sulkowska EM, Xu M, Koibuchi N (2009). Increase in cerebellar neurotrophin-3 and oxidative stress markers in autism. *Cerebellum*.

[42] Chez MG, Dowling T, Patel PB, Khanna P, Kominsky M (2007). Elevation of tumor necrosis factor-alpha in cerebrospinal fluid of autistic children. *Pediatric Neurology*.

[43] Theoharides TC, Asadi S, Patel AB (2013). Focal brain inflammation and autism. *Journal of Neuroinflammation*.

[44] Middleton E, Kandaswami C, Theoharides TC (2000). The effects of plant flavonoids on mammalian cells: implications for inflammation, heart disease, and cancer. *Pharmacological Reviews*.

[45] van Aller GS, Carson JD, Tang W (2011). Epigallocatechin gallate (EGCG), a major component of green tea, is a dual phosphoinositide-3-kinase/mTOR inhibitor. *Biochemical and Biophysical Research Communications*.

[46] Smrž D, Kim M-S, Zhang S (2011). MTORC1 and mTORC2 differentially regulate homeostasis of neoplastic and non-neoplastic human mast cells. *Blood*.

[47] Shang YC, Chong ZZ, Wang S, Maiese K (2011). Erythropoietin and Wnt1 govern pathways of mTOR, Apaf-1, and XIAP in inflammatory microglia. *Current Neurovascular Research*.

[48] Abbott NJ, Rönnbäck L, Hansson E (2006). Astrocyte-endothelial interactions at the blood brain barrier. *Nature Reviews Neuroscience*.

[49] Abbott NJ, Patabendige AAK, Dolman DEM, Yusof SR, Begley DJ (2010). Structure and function of the blood-brain barrier. *Neurobiology of Disease*.

[50] Wolburg H, Wolburg-Buchholz K, Kraus J (2003). Localization of claudin-3 in tight junctions of the blood-brain barrier is selectively lost during experimental autoimmune encephalomyelitis and human glioblastoma multiforme. *Acta Neuropathologica*.

[51] Palmer AM (2011). The role of the blood brain barrier in neurodegenerative disorders and their treatment. *Journal of Alzheimer’s Disease*.

[52] Dhingra D, Parle M, Kulkarni SK (2005). Genetic basis of Alzheimer’s disease. *Indian Journal of Pharmaceutical Sciences*.

[53] Bossy-Wetzel E, Schwarzenbacher R, Lipton SA (2004). Molecular pathways to neurodegeneration. *Nature Medicine*.

[54] Selkoe DJ (2001). Alzheimer’s disease: genes, proteins, and therapy. *Physiological Reviews*.

[55] Rasool M, Malik A, Qazi A (2013). Current view from Alzheimer disease to type 2 diabetes mellitus. *CNS & Neurological Disorders—Drug Targets*.

[56] Felder CC, Bymaster FP, Ward J, DeLapp N (2000). Therapeutic opportunities for muscarinic receptors in the central nervous system. *Journal of Medicinal Chemistry*.

[57] Zhu X, Raina AK, Lee H-G, Casadesus G, Smith MA, Perry G (2004). Oxidative stress signalling in Alzheimer’s disease. *Brain Research*.

[58] Fusco D, Colloca G, Lo Monaco MR, Cesari M (2007). Effects of antioxidant supplementation on the aging process. *Clinical Interventions in Aging*.

[59] Khan RA, Bukhari IA, Nawaz SA, Choudhary MI (2006). Acetylcholinesterase and butyrylcholinesterase inhibitory potential of some Pakistani medicinal plants. *Journal of Basic and Applied Sciences*.

[60] Anilakumar KR, Nagaraj NS, Santhanam K (2007). Reduction of hexachlorocyclohexane-induced oxidative stress and cytotoxicity in rat liver by emblica officinalis gaertn. *Indian Journal of Experimental Biology*.

[61] Kuhn B, Kollman PA (2000). QM-FE and molecular dynamics calculations on catechol O-methyltransferase: free energy of activation in the enzyme and in aqueous solution and regioselectivity of the enzyme-catalyzed reaction. *Journal of the American Chemical Society*.

[62] Lee J-Y, Kim Y (2005). Comparative homology modeling and ligand docking study of human catechol-O-methyltransferase for antiparkinson drug design. *Bulletin of the Korean Chemical Society*.

